# Age-related sarcoma patient experience: results from a national survey in England

**DOI:** 10.1186/s12885-018-4866-8

**Published:** 2018-10-17

**Authors:** Eugenie Younger, Olga Husson, Lindsey Bennister, Jeremy Whelan, Roger Wilson, Andy Roast, Robin L Jones, Winette TA van der Graaf

**Affiliations:** 1 Sarcoma Unit, Royal Marsden Hospital, London, SW3 6JJ UK; 2 Sarcoma UK Registered Cancer Charity, London, N1 6AH UK; 3 University College London Hospital (UCLH), London, NW1 2BU UK; 4 Division of Clinical Studies, Institute of Cancer Research, London, SW7 3RP UK

**Keywords:** Sarcoma, Patient experience, Age-related, Adolescents and young adults, Elderly

## Abstract

**Background:**

Sarcomas are rare, heterogeneous tumours affecting patients of any age. Previous surveys describe that sarcoma patients report a significantly worse experience than those with common cancers. Consequently, Sarcoma UK conducted a national survey and these data were examined for age- and tumour-related differences in patients’ experiences.

**Methods:**

Patients were randomly selected from respondents to National Cancer Patient Experience Surveys (*n* = 900). Differences between patient groups according to age (Adolescents and Young Adults [AYA] 18–39 years, middle-aged 40–64 years, elderly 65 + years) and tumour type (soft-tissue [STS] vs. bone]) were analysed with t-tests or chi-square tests.

**Results:**

Survey response rate was 62% (*n* = 558; STS 75%, bone sarcoma 25%). Delay in diagnosis was reported; 27% patients (*n* = 150) waited > 3 months and initial symptoms were incorrectly interpreted; AYA STS patients were significantly more likely to be treated for another condition, or advised that their symptoms were not serious, than older STS patients. Clinical trial participation was low (6%, *n* = 35). Symptom burden was high, most commonly daytime fatigue (48%, *n* = 277) and pain (44%, *n* = 248). AYAs were significantly more likely to report most side-effects and post-treatment concerns than older patients. Elderly patients were more satisfied with the information and emotional support provided than younger patients, however were significantly less likely to be referred to rehabilitation services.

**Conclusions:**

This study identifies significant age-related differences in the sarcoma patient journey, which are not only related to variation in tumour-types. These results provide rationale for adopting an age-specific approach to the management of sarcoma patients in order to improve overall patient experience.

**Electronic supplementary material:**

The online version of this article (10.1186/s12885-018-4866-8) contains supplementary material, which is available to authorized users.

## Background

Sarcomas are rare and heterogeneous tumours of mesenchymal origin, which account for approximately 1–2% of all adult solid malignancies [1]. In 2010, the incidence of sarcomas in the United Kingdom (UK) was 3829 (3298 soft tissue, 531 bone) [2]. Sarcomas can affect patients of any age and occur at almost any anatomical site. Moreover, there are at least 70 histological subtypes and therefore research is often limited by small patient numbers [1].

Diagnosis is often delayed due to a lack of public awareness of the symptoms of sarcoma, coupled with limited experience among healthcare professionals [3]. Delay may allow the development of advanced disease, which is not amenable to curative surgical resection [1]. In addition, many sarcomas demonstrate an aggressive phenotype and around half of patients with high grade tumours will eventually develop incurable disease [4, 5]. Despite improvements in the prognosis of many other solid malignancies and certain sarcoma subtypes, the five-year overall survival for soft tissue and bone sarcomas remains poor at around 50–60% [6].

Patients with sarcoma often have multiple complex symptoms [7]. Burden of symptoms has been shown to have a negative impact on health-related quality of life (HRQoL) in cancer patients [8, 9]. Patient reported outcomes (PROs) including HRQoL are increasingly recognised as key components of patient-centred care and decision-making [10]. PROs reflect how patients feel and function in relation to a disease, and its treatment, without interpretation by others, including healthcare professionals. PROs enable unique insight into the effectiveness of care from the patient perspective, which has particular relevance in an era of scarce resources and rising treatment-costs [11] In addition, patient self-report can itself improve symptom-management, communication, HRQoL and satisfaction with care [12, 13].

In view of the diversity of sarcoma histotypes across the age-spectrum and specific developmental issues an age-stratified approach to the overall management of sarcomas seems appropriate from both the patients’ and health care professionals’ perspective. Paediatric patients most commonly develop rhabdomyosarcoma, whereas adolescent and young adults (AYA) acquire synovial sarcoma, Ewing sarcoma, osteosarcoma, desmoplastic small round cell tumor, clear cell sarcoma, alveolar soft part sarcoma, epithelioid sarcomas and malignant peripheral nerve sheath tumours [14, 15]. Many of these patients require intensive, multimodal treatment, including chemotherapy, and may face potential long-term side-effects [11]. AYA sarcoma patients also encounter additional psychosocial challenges including changes in body-image and relationships, as well as issues with fertility, higher education, gaining employment and financial difficulties [14, 15]. Adult sarcoma patients most commonly develop undifferentiated sarcomas, gastrointestinal stromal tumours, leiomyosarcomas and liposarcomas requiring surgery, often radiotherapy and on indication chemotherapy [11]. Unlike younger patients, elderly patients often receive less aggressive treatments possibly due to medical co-morbidities; a factor associated with increased mortality [16]. It is also acknowledged that elderly patients are disproportionally underrepresented in clinical research trials [17]. Overall, the treatment of sarcoma patients is often burdensome due to bulky tumours, and at times may be challenging due to anatomic locations such as the head and neck.

In 2014, the UK National Cancer Patient Experience Survey (NCPES) identified that patients with rare cancers, including sarcomas, described a significantly poorer experience of care than those with common cancers [18]. They were less likely to feel that they were seen in a timely manner, among other negative findings [18]. Following these results, Sarcoma UK (the largest sarcoma-specific cancer charity in the UK) commissioned and funded a national survey to gain additional insight into the sarcoma patient journey. The Sarcoma UK data were used in the present study to examine age-related differences in the sarcoma patient journey. Understanding the age-specific patient experience before, during and after treatment is essential in order to develop patient-centered services, which incorporate biomedical and psychosocial needs with existing clinical knowledge.

## Methods

### Patients

**Fig. 1:**
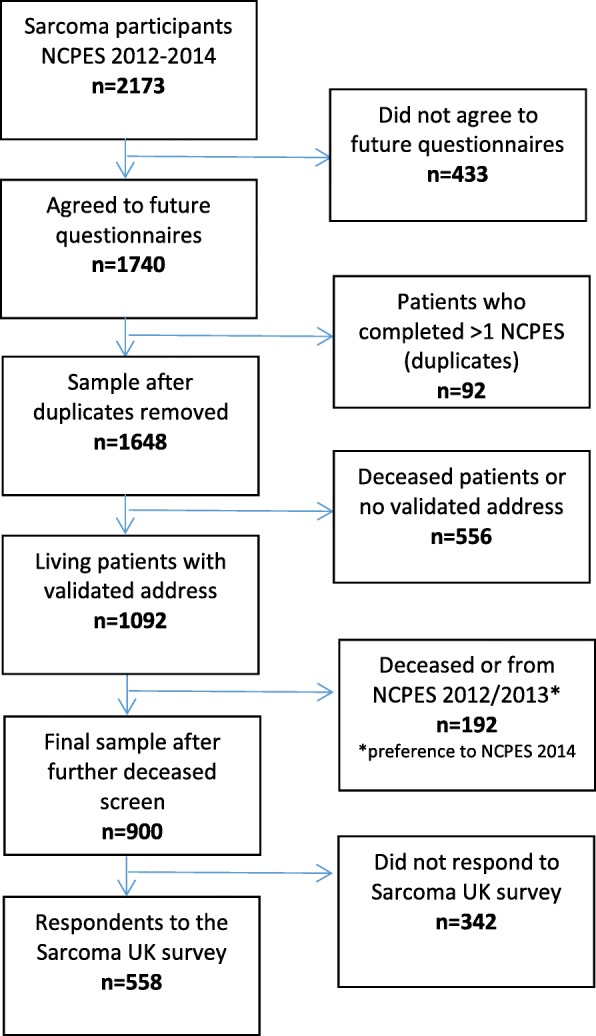
Participant selection process

This study was a cross-sectional survey of adult sarcoma patients (aged 18 years and older) in England. Patients were identified from respondents to the NCPES 2012–14, who indicated willingness to be contacted for future questionnaires. The NCPES is an annual survey commissioned by the National Health Service in England. The NCPES is sent to all patients with cancer who have been discharged after an inpatient or day-case admission (over a specified three-month period) from one of the acute and specialist NHS trusts in England that provide adult cancer services. Public Health England have reported high concurrence between NCPES respondents and a newly diagnosed cancer population-based dataset, with regard to age, sex, ethnicity and deprivation, however NCPES has slight over-representation of patients aged 51–75 years and of white ethnicity: reflecting known bias in survey responders [19]. Patients with tumour types which are more likely to be treated as an outpatient are under-represented [19]. The NCPES does not collect data on date of diagnosis or stage of disease. The flow diagram (Fig. 1) demonstrates how the patient sample was selected for the Sarcoma UK patient survey. Deceased patients, duplicated patients or those without a validated address were identified and excluded from the study. Preference was given to those patients who participated in the most recent NCPES: 2014 (*n* = 452), 2013 (*n* = 343), 2012 (*n* = 105*).* Patients were approached independent of their stage of disease, which was not identified for this survey. The final sample size was 900.

Ethical approval was not required for this secondary study as the NCPES is underpinned by section 251 approval from the Secretary of State to cover every hospital in England providing adult cancer services. Annual approval for NCPES is reviewed by the The Ethics and Confidentiality Committee of the National Information Governance Board. Patient consent was implicit through completion of the Sarcoma UK questionnaire. Patients were advised that they could withdraw the information that they had provided in the questionnaire upon request, up to the point at which personal details were removed and data was analysed.

### Materials

Questionnaires were designed by Quality Health, an independent agency approved by the Care Quality Commission specialising in national patient surveys, in conjunction with Sarcoma UK, patient advocates and sarcoma clinicians. The questionnaire aimed to provide a comprehensive overview of the sarcoma patient experience and focused on three areas of the patient journey: diagnosis, treatment and post-treatment. Questionnaires were dispatched to patients by post and two reminders sent to non-respondents as necessary within the 12-week period. Fieldwork was undertaken between January–March 2015. Questionnaires were returned by post to Quality Health for analysis.

### Statistical analysis

Patients who completed the questionnaire were grouped by tumour-type (STS or bone sarcoma) and age [(AYA; 18–39 years), middle-aged (40–64 years) and elderly (65 + years)]. Certain questions were not applicable to all participants and therefore missing data for these questions was assumed to be ‘missing not at random’ (e.g. question 28 ‘If you had a bone sarcoma were you given information from the Bone Cancer Research trust?’ For other questions only available data were analysed. Missing items were assumed to be missing completely at random. Descriptive statistics were used to describe the study sample. For continuous variables, mean values and standard deviations are presented. Categorical variables are represented by numbers and percentages and chi-square tests were conducted to detect differences between tumour-type and age groups. Adjusted residual values were calculated to identify significant differences with a significance level of α = 0.05. All data analysis was performed using IBM SPSS 22.0.

## Results

### Patient characteristics (Table 1)

Of the 900 patients invited, 558 patients completed the survey (response rate 62%): 418 STS (75%) and 140 bone sarcoma (25%). Response rates for STS patients and bone sarcoma patients were equal (62%). Mean age of respondents was 64.1 years (SD 14, range 18–96). There were 46 AYA (bone sarcoma, *n* = 23, STS *n* = 23), 285 middle-aged (bone sarcoma, *n* = 78, STS *n* = 207) and 227 elderly patients (bone sarcoma *n* = 39, STS *n* = 188). Response rate varied according to age: AYA 60%, middle-aged 82% and elderly 48%. Gender distribution was almost equal (male *n* = 274, 49%, female *n* = 284, 51%). ‘Lower limb’ was the most common primary site of disease in both STS and bone sarcoma patients (total *n* = 177, 32%). The total number of patients treated with surgery was 502 (90%), radiotherapy (*n* = 246 patients, 44.1%) and chemotherapy (*n* = 201, 36.0%). AYA patients (STS and bone sarcoma) were significantly more likely to have been treated with chemotherapy than middle-aged and elderly patients (80% AYA vs. 40% middle-aged vs. 22% elderly: *p* < 0.001). Most patients had three-monthly (*n* = 232, 45.4%) or 6 monthly (*n* = 109, 21.3%) follow-up.

**Table 1:** Patient Demographics

				Bone (*n* = 140)			Soft Tissue (*n* = 418)		
	TOTAL (*n* = 558)	BONE (*n* = 140)	SOFT TISSUE (*n* = 418)	AYA (*n* = 23)	Middle-age (*n* = 78)	Elderly (*n* = 39)	AYA (*n* = 23)	Middle-age (*n* = 207	Elderly (*n* = 188)
Age									
Mean(SD)	64.1 (15.5)	57 (18.0)	66.2 (14.0)	26.4 (6.9)	57.4 (8.7)	77 (5.0)	31.1 (5.6)	59.7 (8.3)	77.6 (5.5)
Gender									
Male	274 (49.1%)	78 (55.7%)	196 (46.9%)	15 (65.2%)	40 (51.3%)	23 (59.0%)	8 (34.8%)	91 (44.0%)	97 (51.6%)
Female	284 (50.9%)	62 (44.3%)	222 (53.1%)	8 (34.8%)	38 (48.7%)	16 (41.0%)	15 (65.2%)	116 (56.0%)	91 (48.4%)
Site of Disease									
Head/neck	70 (12.5%	44 (31.4%)	26 (6.2%)	1 (4.3%)	24 (30.8%)	19 (48.7%)	2 (8.7%)	9 (4.3%)	15 (8.0%)
Thorax	32 (5.7%)	6 (4.3%)	26 (6.2%)	1 (4.3%)	3 (3.8%)	2 (5.1%)	1 (4.3%)	14 (6.8%)	11 (5.9%)
Abdomen	129 (23.1%)	n/a	129 (30.9%)	n/a	n/a	n/a	6 (26.1%)	63 (30.4%)	60 (31.9%)
Pelvis	46 (8.2%)	21 (15.0%)	25 (6.0%)	5 (21.7%)	11 (14.1%)	5 (12.8%)	1 (4.3%)	15 (7.2%)	9 (4.8%)
Upper limb	68 (12.2%)	15 (10.7%)	53 (12.7%)	3 (13.0%)	8 (10.3%)	4 (10.3%)	3 (13.0%)	22 (10.6%)	28 (14.9%)
Lower limb	177 (31.7%)	40 (28.6%)	137 (32.8%)	11 (47.8%)	21 (26.9%)	8 (20.5%)	9 (39.1%)	70 (33.8%)	58 (30.9%)
Vertebral column	6 (1.1%)	6 (4.3%)	n/a	1 (4.3%)	5 (6.4%)	0 (0.0%)	n/a	n/a	n/a
Skin	9 (1.6%)	n/a	9 (2.2%)	n/a	n/a	n/a	1 (4.3%)	6 (2.9%)	2 (1.1%)
Unspecified	21 (3.8%)	8 (5.7%)	13 (3.1%)	1 (4.3%)	6 (7.7%)	1 (2.6%)	0 (0.0%)	8 (3.9%)	
Treatments Received									
Surgery	502 (90.0%)	129 (92.1%)	374 (89.5%)	18 (78.3%)	75 (96.2%)	36 (92.3%)	19 (82.6%)	186 (90.0%)	169 (90%)
Radiotherapy	246 (44.1%)	58 (41.4%)	188 (45.0%)	13 (56.5%)	32 (41.0%)	13 (33.3%)	16 (69.6%)	95 (45.9%)	77 (41.0%)
Chemotherapy	201 (36.0%)	55 (39.3%)	146 (34.9%)	20 (87.0%)	33 (42.3%)	2 (5.1%)	17 (73.9%)	81 (39.1%)	48 (25.5%)
Other	27 (4.8%)	10 (7.1%)	17 (4.1%)	2 (8.7%)	5 (6.4%)	3 (7.7%)	0 (0.0%)	11 (5.3%)	6 (3.2%)
Follow-up									
< 3 monthly	89 (17.4%)	37 (28.2%)	52 (13.7%)	7 (30.4%)	17 (23.9%)	13 (35.1%)	3 (13.4%)	30 (15.8%)	19 (11.2%)
3 monthly	232 (45.4%)	49 (37.4%)	183 (48.2%)	9 (39.1%)	32 (45.1%)	8 (21.6%)	12 (57.1%)	94 (49.5%)	77 (45.6%)
> 3–6 monthly	128 (25.0%)	34 (26.0%)	94 (24.7%)	5 (21.7%)	18 (25.4%)	11 (29.7%)	3 (14.3%)	41 (21.6%)	50 (29.6%)
Annual	23 (4.5%)	6 (4.6%)	17 (4.5%)	2 (8.7%)	1 (1.4%)	3 (0.0%)	0 (0.0%)	11 (5.8%)	6 (3.6%)
Never	12 (2.3%)	1 (0.8%)	11 (2.9%)	0 (0%)	1 (1.4%)	0 (0%)	1 (4.8%)	3 (1.6%)	7 (4.1%)
Variable	27 (5.3%)	4 (3.1%)	23 (6.1%)	0 (0%)	2 (2.8%)	2 (5.4%)	2 (9.5%)	11 (5.8%)	10 (5.9%)

### Diagnosis

The most common presenting symptom overall was a ‘painless lump’ (*n* = 229, 41%). Elderly STS patients were more likely to present with a painless lump than AYA and middle-aged patients (*p* = 0.038). In contrast, bone pain was most common in bone sarcoma patients (*n* = 44, 31%) and was a more common presenting symptom in AYA bone sarcoma patients than other bone sarcoma age groups (*p* = 0.011).

Almost half of patients (*n* = 251, 48%) presented to a medical professional within 4 weeks of first developing symptoms, however, more than a quarter waited > 3 months (*n* = 150, 27%) and one in ten > 1 year (*n* = 54, 10%) before seeking medical advice. Presentation was more likely to be delayed in bone sarcoma than STS patients (*p* = 0.047). There were no age-related differences in time to presentation.

Most patients sought advice from their General Practitioner (GP) (*n* = 447, 80%) about their symptoms. Within this cohort of patients, GPs referred 324 patients (72%) either for further investigations, to a hospital specialist or immediately admitted to hospital. An incorrect interpretation of symptoms and subsequent advice was given to 123 patients (28%). This included those treated for another condition (*n* = 42, 8.6%), those who were told that their symptoms were not serious but to return if they persisted (*n* = 41, 8.4%) and those who were not advised to return if symptoms persisted (*n* = 40, 8.1%). Elderly bone sarcoma patients (*n* = 21, 87.5%) were significantly more likely to be referred for investigation than younger patients while AYA bone sarcoma patients were more frequently treated for another condition or advised their symptoms were not serious (*n* = 10, 50%; *p* = 0.021). Elderly STS patients (*n* = 123, 78.3%) were also more likely to be referred and AYA STS patients more likely to be treated for another condition or advised that their symptoms were not serious (*n* = 8, 53.3%; *p* = 0.001).

Approximately one fifth of patients presented to the Emergency Department (ED) (*n* = 121, 22%). Similarly, of those seen in the ED, 91 patients (75%) were referred for further investigation and 30 patients (25%) were treated for another condition or advised their symptoms were not serious. No significant age-related differences were identified in bone sarcoma patients. However, in STS patients, the elderly were more likely to be further investigated than younger patients and AYA and middle-aged patients were more commonly treated for another condition or told their symptoms were not serious (*p* = 0.02).

Overall, 104 patients (20%) were told that they may have a sarcoma when first presenting with symptoms. This was significantly more likely in elderly bone sarcoma patients (*n* = 15, 45.5%) compared to AYA (*n* = 2, 9.1%) and middle-aged (*n* = 10, 14.3%) bone sarcoma patients (*p* = 0.003), and in elderly STS patients (*n* = 49, 27.7%) compared to AYA (*n* = 4, 17.4%) and middle-aged (*n* = 24, 12.1%) STS patients (*p* = 0.001). For further details, see Additional file 1.

### Treatment

Most patients (*n* = 475, 87%) reported that they were treated by a specialist sarcoma team and had a clinical nurse specialist (*n* = 407, 76%). Patients were often treated at more than one hospital (*n* = 368, 66%). Most patients “did not mind” travelling for surgical treatment (*n* = 451, 89%) and almost half (*n* = 260, 49%) journeyed at least 20 miles for an operation. Patients who were treated with chemotherapy and/or radiotherapy in addition to surgery often had to travel to a different hospital for this treatment (*n* = 209, 62%). AYA bone sarcoma patients (*n* = 17, 85%) were significantly more likely to attend another hospital for these additional treatments (p = < 0.001) and to be treated at more than three hospitals (*p* = 0.033) than older bone sarcoma patients.

One third of patients (*n* = 158, 30%) were offered participation in a clinical trial, of whom a small number participated (*n* = 35, 22%). Consequently, 6% of all participants were involved in a clinical trial. AYA bone sarcoma patients were more likely to be offered (*n* = 13, 57%; *p* = 0.013) and to participate (*n* = 12, 52%; *p* = 0.004) in a clinical trial compared to middle-aged and elderly bone sarcoma patients. There were no significant age-related differences in clinical trial accrual among the STS patients (Additional file 2).

Most patients (*n* = 472, 87%) felt that they were given sufficient information to make informed decisions about their care. Almost half of patients were provided with a complete written treatment plan (*n* = 237, 48%). Elderly patients (bone sarcoma *n* = 39, STS *n* = 188) were more likely to report satisfaction with the information given than AYA and middle-aged patients (bone sarcoma *p* = < 0.001, STS *p* = 0.049).

Most patients (*n* = 441, 81%,) were given adequate emotional support from hospital staff, however 40 patients (7%) would have preferred more support and 64 patients (12%) did not feel that this was necessary. Elderly bone sarcoma patients (*n* = 33, 85%; *p* = 0.006) and elderly STS (*n* = 115, 64%; *p* = 0.003) were more likely to report sufficient emotional support than AYA (*n* = 12, 52%) and middle-aged (*n* = 39, 51%) bone sarcoma patients, and AYA (*n* = 9, 41%) and middle-aged (*n* = 100, 49%) STS patients (Additional file 2).

**Fig. 2:**
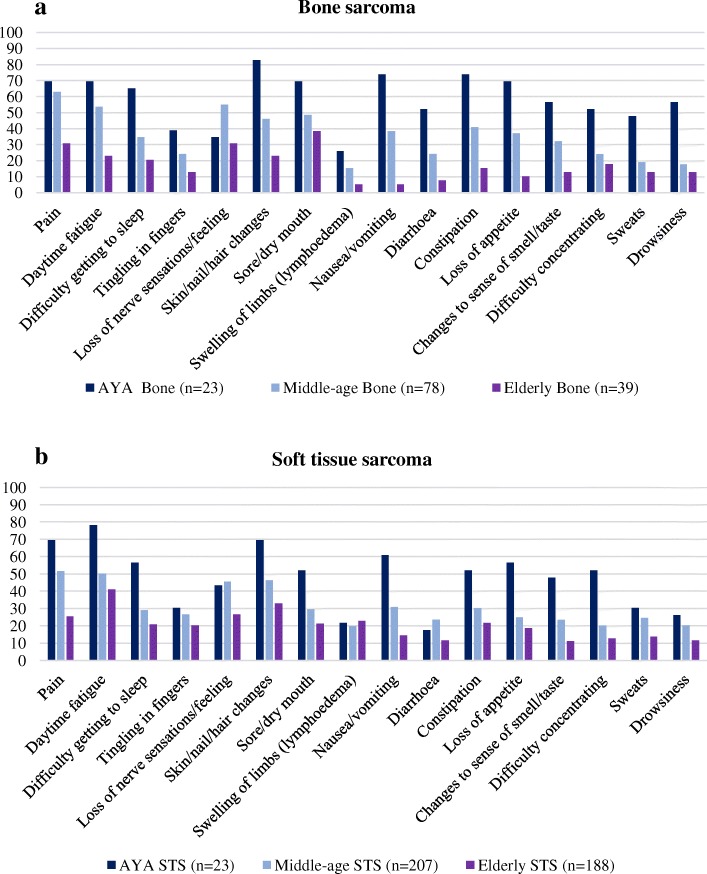
**a**. Percentages of bone sarcoma patients with symptoms or side-effects according to the different age groups. Heading – Bone Sarcoma Legends: • AYA Bone (*n* = 23), •Middle-age Bone (*n* = 78), •Elderly Bone (*n* = 39). **b**. Percentage of soft tissue sarcoma patients with symptoms or side-effects according to the different age groups. Heading – Soft Tissue Sarcoma. Legends: •AYA STS (*n* = 23), •Middle-age STS (*n* = 207), •Elderly (*n* = 188)

The most common symptoms and side effects of treatment were daytime fatigue (*n* = 277, 47.7%) and pain (*n* = 248, 44.4%). Pain was the symptom with the greatest impact on patients’ lives (*n* = 92, 25%). AYA bone sarcoma and STS patients were significantly more likely to recall side effects of treatment than middle-aged and elderly patients. Middle-aged patients were also more likely to report side effects than elderly patients in STS and bone sarcoma groups (Fig. 2a and b).

### Support and follow-up

Most patients felt that follow-up information was very clear (*n* = 428, 85%) and knew how to contact the sarcoma team for information (*n* = 482, 89%). Patients were most likely to be referred to physiotherapy after treatment (*n* = 184, 33%). Referral was significantly more likely in AYA and middle-aged bone sarcoma patients than elderly bone sarcoma patients (*p* = 0.03). Similarly, AYA STS patients were more commonly referred for physiotherapy than older patients (*p* = 0.03). Rehabilitation services were helpful for 176 patients (48%), however 105 patients (29%) found minimal benefit and 49 patients (13%) not useful at all. No age-specific differences were observed.

One quarter of patients (*n* = 133, 24%) were aware of Sarcoma UK and given a Sarcoma UK toolkit (*n* = 104, 19%). A minority of patients were told about generic (*n* = 134, 28%) or sarcoma-specific (*n* = 92, 18%) local support groups. Patients used online national UK charity websites including Macmillan (*n* = 231, 48%) and Cancer Research UK (CRUK) (*n* = 108, 22%). AYA and middle-aged bone sarcoma patients were significantly more likely to use Macmillan (*p* < 0.001) and CRUK (*p* = 0.003) websites than elderly bone sarcoma patients. AYA STS patients were more likely to use Macmillan and Sarcoma UK than older patients (*p* < 0.001). Middle-aged bone sarcoma patients were more likely to use Sarcoma UK than elderly (*p* = 0.032). See Additional file 3.

**Fig. 3:**
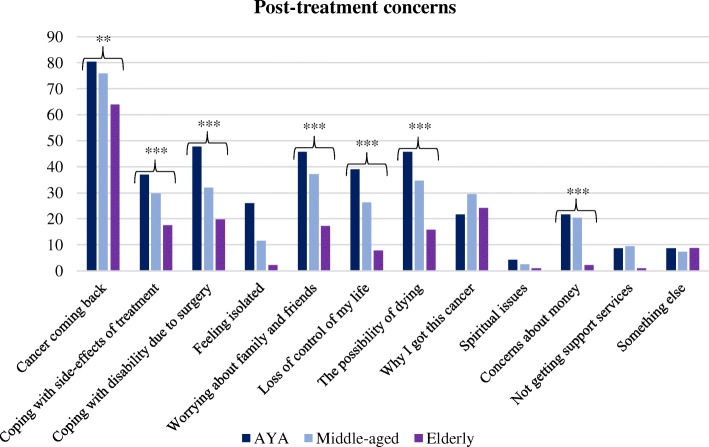
Percentage of sarcoma patients with post-treatment concerns according to age groups. Heading – Post-treatment concerns Legends: •AYA, •Middle-aged, •Elderly

Fear of cancer recurrence was the most common post-treatment concern (*n* = 398, 72%). Many post-treatment concerns were more common in AYA (bone sarcoma and STS patients) compared with middle-aged and elderly patients. Middle-aged patients also reported more worries than elderly patients (Fig. 3: Post-treatment concerns). Worry about ‘cancer coming back’ was significantly more common among AYA and middle-aged patients compared with elderly patients (AYA 80%, middle-aged 76%, elderly 64%, *p* = 0.005). AYA and middle-aged patients were more likely to report worries about ‘coping with side effects of treatment’ (AYA 37%, middle-aged 30%, elderly 18%, *p* = 0.001). AYA were the most likely group to have worries about coping with disability caused by surgery (AYA 48%, middle-aged 32%, elderly 20%, *p* < 0.001). Worries about family and friends were most commonly reported by AYA and middle-aged patients (AYA 46%, middle-aged 37%, elderly 17%, *p* < 0.001). Worries about ‘loss of control of my life’, the ‘possibility of dying’ and financial concerns were also more common in AYA and middle-aged patients compared with elderly patients. There were no other statistically significant differences in worries between age-groups.

## Discussion

To our knowledge, this is the first large-scale study of the sarcoma patient journey from the patient perspective. In accordance with current UK guidelines [20, 21], the majority of patients were treated under the care of a specialist sarcoma team and received support from a clinical nurse specialist.

### Diagnostic trajectory

Delay in patient presentation was observed in the diagnostic trajectory. We found that 27% of patients presented more than 3 months after first noting symptoms and 10% of patients waited more than 1 year before seeking advice for their symptoms. Patient age was not associated with time to presentation. Our findings are consistent with a previous UK survey of almost 2000 cancer patients, which found that 21% of sarcoma patients (*n* = 127) delayed > 3 months from symptom-onset to first presentation, and was not associated with patient-age [22]. Patient-delay in other malignancies varied from 8.5% (breast) to 47.8% (prostate) [22].

Almost three-quarters of participants in this study were referred for further evaluation by their GP or ED doctor, after seeking advice for their symptoms. Approximately one quarter of patients who presented to a medical practitioner with symptoms reported that they were treated for another condition or advised that their symptoms were not serious. AYA patients were more likely to be treated for another condition or advised that their symptoms were not serious, whereas elderly patients were more likely to be referred for further evaluation. Previous data has also demonstrated that AYA cancer patients are significantly more likely to have seen their GP ≥3 times before referral than older patients [23]. This may be due to low clinical suspicion of cancer in this age group and may lead to delay in diagnosis. Overall, given the low incidence of sarcomas, professionals may only encounter one case in their career [24]. In addition, only approximately one in 100 soft tissue lumps are malignant, leading to low clinical suspicion [24]. Previous research has shown that patients with sarcomas are more likely to have seen their GP > 2 times before referral to hospital (41%) compared to others malignancies, such as breast 8% and skin 10% cancer [18]. They are also less likely to be referred by the ‘Two Week Wait’ (TWW) system, which ensures that patients with suspected cancer are seen within 2 weeks (10% bone sarcoma, 12% STS vs. 25% all cancers) [25]. Patients with central nervous system (1%) and pancreatic (11%) malignancies are also less commonly diagnosed via the TWW route [26].

We found that one fifth of patients presented with first symptoms to the ED but this was not associated with patient age. The high number of emergency presentations is particularly concerning as this is associated with more advanced disease and significantly lower survival rates in patients with other cancers, even when adjusted for stage of disease at diagnosis [25–27]. A previous UK study demonstrated that 18.6% of sarcomas and 23% of all cancers were diagnosed through ‘emergency presentation’ between 2006 and 2008 [25]. This study also found that bone sarcoma patients aged < 10 or > 80 years, and STS patients aged < 19 or > 80, were more likely to present to ED [25]. Although we did not find this association, our survey did not include paediatric patients and our sample size was considerably smaller (558 vs. 8956 patients) [25]. Others have also found that emergency presentation is more common in elderly cancer patients, which may itself contribute to increased mortality [26]. Emergency presentation may be unavoidable due to tumour-related factors; however research indicates that many patients have had consultations within 12 months of diagnosis. This indicates that there may be opportunities to reduce the number of emergency cancer presentations [27].

UK cancer survival figures are significantly lower than most European countries, a finding that has been attributed to late stage diagnosis [28]. In the UK a relatively high number of patients present to the ED before diagnosis [27]. The UK Royal College of Emergency Medicine (RCEM) and The Patients Association reported that patients often attended ED because they felt unable to access timely help elsewhere and perceived that they would be able to access immediate diagnostic investigations and specialist opinion [29]. This included patients with symptoms which had been present for days or even weeks, who were offered a GP appointment the same day however still chose to attend ED instead. For lung cancer this has been exemplified by a study of eight European countries. In this study an emergency route to lung cancer diagnosis was most likely in the UK and Portugal, and least common in The Netherlands [30]. Disparities in routes to diagnosis may influence outcomes for UK cancer patients compared with their European counterparts.

### Treatment

In accordance with findings of the NCPES, less than one third of patients (30%) were offered the opportunity to participate in a clinical trial [18]. AYA bone sarcoma patients were significantly more likely to be involved in a clinical trial than all other groups. These findings reflect the availability of clinical trials enrolling patients at the time of the survey, which included large international trials for bone sarcoma and few trials for first presentation of STS. It is internationally acknowledged that there are significant age inequalities in clinical trial accrual among cancer patients. Previous studies have demonstrated that AYA cancer patients overall are less commonly enrolled in clinical trials than paediatric and adult patients [31]. Lack of involvement in research trials is believed to be responsible for slower progress in survival outcomes for AYA cancer patients [31, 32]. Older patients are also often disproportionally underrepresented in clinical research trials despite an increasing incidence of sarcomas with advancing age [33].

Comparable with other cancers, many sarcoma patients reported a high burden of symptoms and side-effects of treatment, most commonly daytime fatigue [34]. Notably, almost all symptoms and side effects of treatment occurred with increased frequency among AYA patients, however AYAs were significantly more likely to have been treated with chemotherapy than older patients. A large study of patients with advanced cancer also found that increasing age was associated with lower reporting of symptoms, however this association has not been replicated in all studies [35]. Older patients may report less symptoms due to physiological changes associated with ageing, lower levels of physical activity, less work-related and social activities compared with younger patients, and possibly a more tolerant attitude toward illness [35, 36]. Elderly patients were significantly more satisfied with the information and emotional support provided than younger patients. This is consistent with research demonstrating a paternalistic attitude of older patients, who report higher levels of satisfaction with care, which correlates with enhanced QoL than younger patients [37].

### Supportive care

The majority of post-treatment concerns were most common in AYA patients. AYAs who are diagnosed with cancer are suddenly faced with existential questions during a critical phase in their life [32]. While their siblings and peers are progressing forward with education and relationships, their own journey is suddenly and unexpectedly altered [32]. The AYA-HOPE study also found a high prevalence of unmet psychosocial needs and reduced HRQoL in AYA cancer survivors compared with an age and sex matched population [38]. Studies of multiple myeloma and lymphoma patients have also demonstrated that the elderly are less likely to experience deterioration in HRQoL, compared with a normative population, than younger patients [39, 40].

### Clinical implications

Despite the growing body of evidence that early diagnosis improves survival in many common cancers, the impact in sarcomas has not been clearly established and may be limited to certain subtypes [24, 41–43]. The intrinsically aggressive disease biology of some sarcomas may override any potential influence of delay on survival outcomes [32]. Nonetheless, it would seem rational to strive toward early diagnosis for those in whom early treatment may improve outcome. Additionally, delayed diagnosis can cause psychological distress and may lead to tumour growth with reduced potential for curative localised surgery. Public awareness campaigns for common cancers are well established. Strategies to improve awareness of ‘alarm’ symptoms of sarcomas, such as a lump larger than a ‘golf ball’, increasing size > 5 cm, deep to the fascia, or persistent deep bone pain have recently been introduced to public awareness programs [44, 45]. Medical professionals must also be aware that sarcomas can affect AYAs and thus avoid delayed referral in this age group. Unlike care for paediatric patients, which is often centralized and protocol-driven, AYAs often fall between services for paediatric and adult patients [46]. AYAs also encounter unique developmental challenges and it is unquestionable that an age-specific approach is required for these patients [47].

The high symptom-burden must be addressed, particularly for AYAs and those who have received intensive treatment. Early integration of specialist symptom control teams can improve HRQoL, satisfaction with care and reduce rates of depression in cancer patients [48]. In addition, this intervention has demonstrated a survival benefit in metastatic non-small-cell lung cancer patients [49]. Pain screening and early referral to a pain treatment protocol has been successful in head and neck cancer patients [50]. Based on results from this survey, it is probable that a multidisciplinary, symptom-oriented approach would be also beneficial for the sarcoma population.

Elderly patients with bone sarcoma were uncommonly involved in research and overall were less likely to be referred to rehabilitation services. Although physical and cognitive problems may require optimisation, it is vital that chronological age is not a barrier to involvement in clinical research, nor interventions which may improve HRQoL [17].

### Limitations

Limitations of this study include the lack of patient details, such as race or ethnicity, geographic location of participants, disease stage and interval from diagnosis to participation in the study. Patients with localized disease encounter very different challenges to those with advanced disease, and consequently, the lack of data regarding disease stage means that it is not clear whether this sample is truly representative of the general sarcoma population. Additionally, the NCPES only includes patients who were discharged from hospital following a day-case or inpatient admission. This means that patients with certain sarcoma subtypes, such as endometrial stromal sarcoma or gastro-intestinal stromal tumour (GIST), who are more commonly treated as an outpatient with oral therapy would not have been invited to participate in the NCPES and thus are likely to be under-represented in the Sarcoma UK patient survey.

Age-based comparisons were limited due to the small sample size of the group of AYA patients (*n* = 46), however statistical analysis was used to identify true differences across the age groups. The response rate was higher in middle-aged patients than AYA or elderly patients: reflecting a known bias in survey respondents. Survivorship bias was introduced by selection of patients who participated in NCPES from 2012 to 2014, which means they will have survived at least 1 year since diagnosis. These patients are likely to have a better prognosis than an unselected population of sarcoma patients. This study did not use internationally a validated questionnaire, however was designed by sarcoma patients and experts in a similar format to previous NCPES in order that patients were familiar with the terminology used. The meaning of a ‘clinical trial’ was open to interpretation by the patient and therefore participation rates should be interpreted with caution. As with all patient surveys, it is possible that recall bias had an influence on our results. The perspective of the healthcare professional (e.g. GP) was not surveyed and therefore all results are based on the patient’s perception of their individual experience.

## Conclusions

In this study, describing the largest sarcoma patient survey to date, we identified significant age-related differences in the sarcoma patient journey. AYA patients appear more vulnerable to incorrect diagnosis, a high burden of treatment related side effects and post-treatment psychological concerns. Elderly patients reported less trial participation and were less likely to be referred to rehabilitation services. Overall, the burden of disease was felt high. Despite the general focus of physicians and researchers on the anatomic and histological heterogeneity of sarcomas, from a patient perspective attention should also be paid to the differences in age-related aspects of diagnosis, treatment, trial access and survivorship. The findings suggest a rationale for integrating an age-stratified approach to the general management of sarcoma patients.

## Additional files

Additional file 1: Diagnosis: data on presenting symptoms, time to presentation, GP and ED action and information about diagnosis for STS and bone sarcomas. (PDF 263 kb)

Additional file 2: Treatment: data on treatment in sarcoma specialist team, no of hospitals attended, travel distance to surgery, chemo/radiotherapy, clinical trials, clinical nurse specialist, information of treatment plan, emotional support. (PDF 436 kb)

Additional file 3: Support and Follow-up: data on support sought and used, post-treatment concerns and clarity of follow-up information. (PDF 250 kb)

## Abbreviations

AYA: Adolescents and Young Adults
CRUK: Cancer Research UK
ED: Emergency department
GIST: Gastro-intestinal stromal tumour
GP: General practitioner
HRQoL: Health-related quality of life
NCPES: UK National Cancer Patient Experience Survey
PROs: Patient reported outcomes
RCEM: Royal College of Emergency Medicine
STS: Soft Tissue sarcomas
TWW: Two Week Wait’
UK: United Kingdom
