# Supplementary material for: Age-related sarcoma patient experience: results from a national survey in England
Source: BMC Cancer. 2018 Oct 17;18:991. doi: 10.1186/s12885-018-4866-8 (PMC6192120; doi:10.1186/s12885-018-4866-8)
Supplement: Supplementary file 1 — Diagnosis: data on presenting symptoms, time to presentation, GP and ED action and information about diagnosis for STS and bone sarcomas. (PDF 263 kb) [file 12885_2018_4866_MOESM1_ESM.pdf]

Supplementary Material: DIAGNOSIS												
						BONE (n=140)				SOFT TISSUE		
	TOTAL (n=558)	BONE (n=140)	SOFT TISSUE (n=418)	p-value	AYA (n=23)	Middle-age (n=78)	Elderly (n=39)	p-value	AYA (16-34)	Middle-age (35 -64)	Elderly (>65)	p-value
**Presenting Symptoms**												
Painless lump	229 (41.0%)	39 (27.9%)	190 (45.5%)	**<0.001**	4 (17.4%)	22 (28.2%)	13 (33.3%)	0.398	5 (21.7%)	92 (44.4%)	93 (49.5%)	**0.038**
Painful Lump	81 (14.5%)	21 (15.0%)	60 (14.4%)	0.851	6 (26.1%)	13 (16.7%)	2 (5.1%)	0.068	5 (21.7%)	32 (15.5%)	23 (12.2%)	0.384
Lump increasing in size	166 (29.7%)	30 (21.4%)	136 (32.5%)	**0.013**	3 (13.0%)	18 (23.1%)	9 (23.1%)	0.563	7 (30.4%)	62 (30%)	67 (35.6%)	0.472
New lump where lump previously removed	34 (6.1%)	8 (5.7%)	26 (6.2%)	0.829	0 (0.0%)	5 (6.4%)	3 (7.7%)	0.417	1 (4.3%)	16 (7.7%)	9 (4.8%)	0.447
Bone pain	56 (10.0%)	44 (31.4%)	12 (2.9%)	**<0.001**	13 (56.5%)	23 (29.5%)	8 (20.5%)	**0.011**	1 (4.3%)	7 (3.4%)	4 (2.1%)	0.689
Something else	173 (31.0%)	36 (25.7%)	137 (32.8%)	0.118	4 (11.1%)	24 (66.7%)	8 (20.5%)	0.297	10 (43.5%)	70 (33.8%)	57 (30.3%)	0.404
**Time to Presentation**												
<2 weeks	140 (25.8%)	26 (19.4%)	114 (27.9%)		6 (27.3%)	13 (17.6%)	7 (18.4%)		7 (30.4%)	60 (30.0%)	47 (25.4%)	
>2 weeks <4 weeks	111 (20.5%)	23 (17.2%)	88 (21.6%)		6 (27.3%)	8 (10.8%)	9 (23.7%)		9 (39.1%)	35 (17.5%)	44 (23.8%)	
>4 weeks <3 months	117 (21.6%)	33 (24.6%)	84 (20.6%)		3 (13.6%)	21 (28.4%)	9 (23.7%)		1 (4.3%)	42 (21%)	41 (22.2%)	
>3 months <6 months	54 (10.0%)	13 (9.7%)	41 (10.0%)		4 (18.2%)	6 (8.1%)	3 (7.9%)		2 (8.7%)	22 (11%)	17 (9.2%)	
>6 months <1 year	42 (7.7%)	9 (6.7%)	33 (8.1%)		2 (9.1%)	7 (9.5%)	0 (0.0%)		3 (13.0%)	14 (7%)	16 (8.6%)	
> 1 year	54 (10.0%)	21 (15.7%)	33 (8.1%)		1 (4.5%)	15 (20.3%)	5 (13.2%)		0 (0%)	19 (9.5%)	14 (7.6%)	
Unsure	24 (4.4%)	9 (6.7%)	15 (3.7%)		0 (0.0%)	4 (5.4%)	5 (13.2%)		1 (4.3%)	8 (4%)	6 (3.2%)	
				**0.047**				0.094				0.353
**GP Action***												
Referred for tests	153 (31.2%)	39 (31.2%)	114 (31.1%)		6 (27.3%)	23 (31.9%)	10 (32.3%)		6 (33.3%)	61 (33.0%)	47 (28.8%)	
Referred to specialist	148 (20.1%)	33 (26.4%)	115 (31.4%)		4 (18.2%)	19 (26.4%)	10 (32.3%)		0 (0.0%)	48 (25.9%)	67 (41.1%)	
Sent to hospital same day	23 (4.7%)	3 (2.4%)	20 (5.5%)		0 (0.0%)	2 (2.8%)	1 (3.2%)		1 (5.6%)	10 (5.4%)	9 (5.5%)	
Treated for another condition	42 (8.6%)	10 (8.0%)	32 (8.7%)		1 (4.5%)	7 (9.7%)	2 (6.5%)		3 (16.7%)	19 (10.3%)	10 (6.1%)	
Told not serious, not told to come back	40 (8.1%)	9 (7.2%)	31 (8.5%)		4 (18.2%)	4 (5.6%)	1 (3.2%)		0 (0.0%)	17 (9.2%)	14 (8.6%)	
Told not serious but told to come back if continued	41 (8.4%)	10 (8.0%)	31 (8.5%)		5 (22.7)	5 (6.9%)	0 (0.0%)		5 (27.8%)	16 (8.6%)	10 (6.1%)	
Didn't go to GP	44 (9.0%)	21 (16.8%)	23 (6.3%)		2 (9.1%)	12 (16.7%)	7 (22.6%)		3 (16.7%)	14 (7.6%)	6 (3.7%)	
				**0.025**				0.149				**0.001**
Referred for tests/specialist/hospital	324 (72%)	75 (72.1%)	249 (72.5%)		10 (50%)	44 (73.3%)	21 (87.5%)		7 (46.7%)	119 (69.6%)	123 (78.3%)	
Treated for another condition/told not serious	123 (28%)	29 (27.9%)	94 (27.4%)		10 (50%)	16 (26.7%)	3 (12.5%)		8 (53.3%)	52 (30.4%)	34 (21.7%)	
				0.924				**0.021**				**0.015**
**Emergency Department (ED) Action***												
Referred for tests	62 (13.2%)	19 (16.0%)	43 (12.3%)		6 (28.6%)	7 (10.6%)	6 (18.8%)		6 (26.1%)	12 (7.0%)	25 (16.0%)	
Referred to specialist	29 (6.2%)	5 (4.2%)	24 (6.9%)		0 (0.0%)	5 (7.6%)	0 (0.0%)		1 (4.3%)	10 (5.8%)	13 (8.3%)	
Treated for another condition	12 (2.6%)	1 (0.8%)	11 (3.1%)		0 (0.0%)	1 (1.5%)	0 (0.0%)		1 (4.3%)	8 (4.7%)	2 (1.3%)	
Told not serious, not told to come back	13 (2.8%)	5 (4.2%)	8 (2.3%)		0 (0.0%)	2 (3.0%)	3 (9.4%)		3 (13.0%	4 (2.3%)	1 (0.6%)	
Told not serious but told to come back if continued	5 (1.1%)	2 (1.7%)	3 (0.9%)		2 (9.5%)	0 (0.0%)	0 (0.0%)		1 (4.3%)	0	2 (1.3%)	
Didn't go to ED	348 (74.2%)	87 (73.1%)	261 (74.6%)		13 (61.9%)	51 (77.3%)	23 (71.9%)		11 (47.8%)	137 (80.1%)	113 (72.4%)	
				0.355				**0.019**				**<0.001**
Referred for tests/specialist/hospital	91 (75%)	24 (75.0%)	67 (75.3%)		6 (75%)	12 (80%)	6 (66.7%)		7 (58.3%)	22 (64.7%)	38 (88.4%)	
Treated for another condition/told not serious	30 (25%)	8 (25.0%)	22 (24.7%)		2 (25%)	3 (20%)	3 (33.3%)		5 (41.7%)	12 (35.3%)	5 (11.6%)	
				0.975				0.766				**0.02**
**Told Sarcoma was possible diagnosis***												
Yes	104 (19.4%)	27 (21.6%)	77 (19.3%)		2 (9.1%)	10 (14.3%)	15 (45.5%)		4 (17.4%)	24 (12.1%)	49 (27.7%)	
No	420 (78.5%)	98 (78.4%)	322 (80.7%)		20 (90.9%)	60 (85.7%)	18 (54.5%)		19 (82.6%)	175 (87.9%)	128 (72.3%)	
				0.573				**<0.001**				**0.001**

***Patient's perception - GP/ED perspective not surveyed.**
