# Supplementary material for: Age-related sarcoma patient experience: results from a national survey in England
Source: BMC Cancer. 2018 Oct 17;18:991. doi: 10.1186/s12885-018-4866-8 (PMC6192120; doi:10.1186/s12885-018-4866-8)
Supplement: Supplementary file 2 — Treatment: data on treatment in sarcoma specialist team, no of hospitals attended, travel distance to surgery, chemo/radiotherapy, clinical trials, clinical nurse specialist, information of treatment plan, emotional support. (PDF 436 kb) [file 12885_2018_4866_MOESM2_ESM.pdf]

SUPPLEMENTARY MATERIAL: TREATMENT												
						BONE (n=140)				SOFT TISSUE (n=418)		
	TOTAL (n=558)	BONE (n=140)	SOFT TISSUE (n=418)	p-value	AYA (n=23)	Middle-age (n=78)	Elderly (n=39)	p-value	AYA (n=23)	Middle-age (n=207)	Elderly (n=188)	p-value
**Treated by a Specialist Sarcoma team**												
Yes	475 (87.2%)	122 (87.8%)	353 (86.9%)		19 (82.6%)	67 (87.0%)	36 (92.3%)		19 (82.6%)	167 (83.1%)	167 (91.8%)	
Not sure	55 (10.1%)	12 (8.6%)	43 (10.6%)		2 (8.7%)	8 (10.4%)	2 (5.1%)		3 (13.0%)	30 (14.9%)	10 (5.5%)	
No	15 (2.8%)	5 (3.6%)	10 (2.5%)		2 (8.7%)	2 (2.6%)	1 (2.6%)		1 (4.3%)	4 (2.0%)	5 (2.7%)	
				0.642				0.560				**0.047**
**No Hospitals attended for treatment**												
1	185 (33.6%)	53 (38.4%)	132 (32.0%)		4 (17.4%)	28 (36.8%)	21 (53.8%)		10 (43.5%)	56 (27.6%)	66 (35.5%)	
2	249 (45.3%)	56 (40.6%)	193 (46.8%)		9 (39.1%)	35 (46.1%)	12 (30.8%)		9 (39.1%)	100 (49.3%)	84 (45.2%)	
3	84 (15.3%)	16 (11.6%)	68 (16.5%)		5 (21.7%)	7 (9.2%)	4 (10.3%)		4 (17.4%)	32 (15.4%)	32 (17.2%)	
>3	32 (5.8%)	13 (9.4%)	19 (4.6%)		5 (21.7%)	6 (7.9%)	2 (5.1%)		0 (0.0%)	15 (7.4%)	4 (2.2%)	
				**0.048**				**0.033**				0.102
**Distance travelled for surgery**												
<5 miles	58 (11.7%)	9 (7.0%)	49 (13.4%)		1 (5.0%)	5 (6.8%)	3 (8.6%)		4 (21.1%)	23 (12.6%)	22 (13.3%)	
5-10 miles	66 (13.4%)	15 (11.7%)	51 (13.9%)		1 (5.0%)	8 (11.0%)	6 (17.1%)		1 (5.3%)	32 (17.6%)	18 (10.9%)	
10-20 miles	110 (22.3%)	22 (17.2%)	88 (24.0%)		2 (10.0%)	12 (16.4%)	8 (22.9%)		6 (31.6%)	42 (23.1%)	40 (24.2%)	
20-50 miles	142 (28.7%)	36 (28.1%)	106 (29.0%)		7 (35.0%)	22 (30.1%)	7 (20.0%)		4 (21.1%)	50 (27.3%)	52 (31.5%)	
>50 miles	118 (23.9%)	46 (35.9%)	72 (19.7%)		9 (45.0%)	26 (35.6%)	11 (31.4%)		4 (21.1%)	35 (19.2%)	33 (20.0%)	
				**0.003**				0.744				0.620
**Did not mind travelling for surgery**												
	451 (88.8%)	118 (88.7%)	333 (88.8%)	0.980	17 (89.5%)	66 (86.8%)	35 (92.1%)	0.700	19 (95%)	165 (88.2%)	149 (88.7%)	0.659
**Chemo/radiotherapy given in different hospital to surgery.**												
	209 (61.8%)	53 (65.4%)	156 (60.7%)	0.445	17 (89.5%)	30 (65.2%)	6 (37.5%)	**<0.001**	11 (64.7%)	85 (62.5%)	60 (57.7%)	0.707
**Offered clinical trial**												
Yes	158 (29.9%)	39 (29.3%)	119 (30.1%)		14 (60.9%)	17 (23.0%)	8 (22.2%)		6 (27.3%)	66 (33.2%)	47 (26.9%)	
No	324 (61.2%)	86 (64.7%)	238 (60.1%)		9 (39.1%)	51 (68.9%)	26 (72.2%)		13 (59.1%)	112 (56.3%)	113 (64.6%)	
Can't remember	47 (8.9%)	8 (6.0%)	39 (9.8%)		0 (0%)	6 (8.1%)	2 (5.6%)		3 (13.6%)	21 (10.6%)	15 (8.6%)	
				0.367				**0.007**				0.546
**Took part in clinical trial**												
	116 (22.2%)	33 (25.0%)	83 (21.3%)	0.374	12 (52.2%)	13 (18.3%)	8 (21.1%)	**0.004**	4 (19.0%)	46 (24%)	33 (18.6%)	0.445
**Clinical nurse specialist**												
Yes, same	338 (63.4%)	83 (62.9%)	255 (63.6%)		17 (77.3%)	40 (54.1%)	26 (72.2%)		14 (63.6%)	116 (58.3%)	125 (69.4%)	
Sometimes	68 (12.8%)	21 (15.9%)	47 (11.7%)		1 (4.5%)	15 (20.3%)	5 (13.9%)		3 (13.6%)	28 (14.1%)	16 (8.9%)	
No	79 (14.8%)	18 (13.6%)	61 (15.2%)		3 (13.6%)	10 (13.5%)	5 (13.9%)		2 (9.1%)	36 (18.1%)	23 (12.8%)	
Don't know	48 (9.0%)	10 (7.6%)	38 (9.5%)		1 (4.5%)	9 (12.2%)	0 (0.0%)		3 (13.6%)	19 (9.5%)	16 (8.9%)	
				0.585				0.127				0.344
**Sufficient information to make decisions on care**												
Yes, all	472 (86.6%)	116 (84.1%)	356 (87.5%)		19 (82.6%)	60 (77.9%)	37 (97.4%)		19 (86.4%)	166 (82.6%)	171 (92.9%)	
Only some	63 (11.6%)	19 (13.8%)	44 (10.8%)		1 (4.3%)	17 (22.1%)	1 (2.6%)		3 (13.6%)	30 (14.9%)	11 (6.0%)	
No	6 (1.1%)	1 (0.7%)	5 (1.2%)		1 (4.3%)	0 (0%)	0 (0%)		0 (0%)	4 (2.0%)	1 (0.5%)	
Did not want to be involved	4 (0.7%)	2 (1.4%)	2 (0.5%)		2 (8.7%)	0 (0%)	0 (0%)		0 (0%)	1 (0.5%)	1 (0.5%)	
				0.485				**<0.001**				0.105
**Given a written treatment plan**												
	300 (61.3%)	81 (64.3%)	219 (60.3%)	0.432	16 (69.6%)	40 (58.8%)	25 (71.4%)	0.379	12 (54.5%)	101 (57.7%)	106 (63.9%)	0.434
**Given enough emotional support**												
Yes, definitely	308 (56.5%)	84 (60.9%)	224 (55.0%)		12 (52.2%)	39 (51.3%)	33 (84.6%)		9 (40.9%)	100 (48.8%)	115 (63.9%)	
Yes, to some extent	133 (24.4%)	32 (23.2%)	101 (24.8%)		8 (34.8%)	21 (27.6%)	3 (7.7%)		10 (45.5%)	55 (26.8%)	36 (20.0%)	
No, would have liked more	40 (7.3%)	11 (8.0%)	29 (7.1%)		3 (13.0%)	8 (10.5%)	0 (0%)		3 (13.6%)	19 (9.3%)	7 (3.9%)	
Did not need	64 (11.7%)	11 (8.0%)	53 (13.0%)		0 (0%)	8 (10.5%)	3 (7.7%)		0 (0%)	31 (15.1%)	22 (12.2%)	
				0.381				**0.006**				**0.003**

Supplementary Material: TREATMENT												
						BONE (n=140)				SOFT TISSUE (n=418)		
	TOTAL (n=558)	BONE (n=140)	SOFT TISSUE (n=418)	p-value	AYA (n=23)	Middle-age (n=78)	Elderly (n=39)	p-value	AYA (n=23)	Middle-age (n=207)	Elderly (n=188)	p-value
**Symptoms/side-effects of treatment**												
Pain	**248 (44.4%)**	77 (55.0%)	171 (40.9%)	**0.004**	16 (69.6%)	49 (62.8%)	12 (30.8%)	**0.001**	16 (69.6%)	107 (51.7%)	48 (25.5%)	**<0.001**
Nausea/vomiting	**154 (27.6%)**	49 (35.0%)	105 (25.1%)	**0.024**	17 (73.9%)	30 (38.5%)	2 (5.1%)	**<0.001**	14 (60.9%)	64 (30.9%)	27 (14.4%)	**<0.001**
Diarrhoea	**109 (19.5%)**	34 (24.3%)	75 (17.9%)	0.101	12 (52.2%)	19 (24.4%)	3 (7.7%)	**<0.001**	4 (17.4%)	49 (23.7%)	22 (11.7%)	**0.008**
Constipation	**171 (30.6%)**	55 (39.3%)	116 (27.8%)	**0.01**	17 (73.9%)	32 (41.0%)	6 (15.4%)	**<0.001**	12 (52.2%)	63 (30.4%)	41 (21.8%)	**0.004**
Skin/nail/hair changes	**238 (42.7%)**	64 (45.7%)	174 (41.6%)	0.397	19 (82.6%)	36 (46.2%)	9 (23.1%)	**<0.001**	16 (69.6%)	96 (46.4%)	62 (33.0%)	**0.001**
Sore/dry mouth	**182 (32.6%)**	69 (49.3%)	113 (27.0%)	**<0.001**	16 (69.6%)	38 (48.7%)	15 (38.5%)	0.06	12 (52.2%)	61 (29.5%)	40 (21.3%)	**0.004**
Tingling in fingers	**133 (23.8%)**	33 (23.6%)	100 (23.9%)	0.933	9 (39.1%)	19 (24.4%)	5 (12.8%)	0.06	7 (30.4%)	55 (26.6%)	38 (20.2%)	0.252
Loss of nerve sensations/feeling	**217 (38.9%)**	63 (45.0%)	154 (36.8%)	0.087	8 (34.8%)	43 (55.1%)	12 (30.8%)	**0.025**	10 (43.5%)	94 (45.4%)	50 (26.6%)	**<0.001**
Loss of appetite	**149 (26.7%)**	49 (35.0%)	100 (23.9%)	**0.01**	16 (69.6%)	29 (37.2%)	4 (10.3%)	**<0.001**	13 (56.5%)	52 (25.1%)	35 (18.6%)	**<0.001**
Changes to sense of smell/taste	**124 (22.2%)**	43 (30.7%)	81 (19.4%)	**0.005**	13 (56.5%)	25 (32.1%)	5 (12.8%)	**0.001**	11 (47.8%)	49 (23.7%)	21 (11.2%)	**<0.001**
Difficulty concentrating	**116 (20.8%)**	38 (27.1%)	78 (18.7%)	**0.032**	12 (52.2%)	19 (24.4%)	7 (17.9%)	**0.01**	12 (52.2%)	42 (20.3%)	24 (12.8%)	**<0.001**
Difficulty getting to sleep	**162 (29.0%)**	50 (35.7%)	112 (26.8%)	**0.044**	15 (65.2%)	27 (34.6%)	8 (20.5%)	**0.002**	13 (56.5%)	60 (29.0%)	39 (20.7%)	**0.001**
Swelling of limbs (lymphoedema)	**109 (19.5%)**	20 (14.3%)	89 (21.3%)	0.07	6 (26.1%)	12 (15.4%)	2 (5.1%)	0.068	5 (21.7%)	41 (19.8%)	43 (22.9%)	0.758
Sweats	**115 (20.6%)**	31 (22.1%)	84 (20.1%)	0.604	11 (47.8%)	15 (19.2%)	5 (12.8%)	**0.004**	7 (30.4%)	51 (24.6%)	26 (13.8%)	**0.012**
Drowsiness	**102 (18.3%)**	32 (22.9%)	70 (16.7%)	0.105	13 (56.5%)	14 (17.9%)	5 (12.8%)	**<0.001**	6 (26.1%)	42 (20.3%)	22 (11.7%)	**0.034**
Daytime fatigue	**266 (47.7%)**	67 (47.9%)	199 (47.6%)	0.959	16 (69.6%)	42 (53.8%)	9 (23.1%)	**0.001**	18 (78.3%)	104 (50.2%)	77 (41.0%)	**0.002**
Something else	**49 (8.8%)**	14 (10.0%)	35 (8.4%)	0.556	2 (8.7%)	9 (11.5%)	3 (7.7%)	0.787	5 (21.7%)	16 (7.7%)	14 (7.4%)	0.058
Don't know	**19 (3.4%)**	2 (1.4%)	17 (4.1%)	0.136	0 (0%)	1 (1.3%)	1 (2.6%)	0.704	1 (4.3%)	6 (2.9%)	10 (5.3%)	0.476
