# Supplementary material for: Age-related sarcoma patient experience: results from a national survey in England
Source: BMC Cancer. 2018 Oct 17;18:991. doi: 10.1186/s12885-018-4866-8 (PMC6192120; doi:10.1186/s12885-018-4866-8)
Supplement: Supplementary file 3 — Support and Follow-up: data on support sought and used, post-treatment concerns and clarity of follow-up information. (PDF 250 kb) [file 12885_2018_4866_MOESM3_ESM.pdf]

Supplementary Material: SUPPORT & FOLLOW-UP												
						BONE (n=140)				SOFT TISSUE (n=418)		
	TOTAL (n=558)	BONE (n=140)	SOFT TISSUE (n=418)	p-value	AYA (n=23)	Middle-age (n=78)	Elderly (n=39)	p-value	AYA (n=23)	Middle-age (n=207)	Elderly (n=188)	p-value
**Post-treatment concerns**												
Cancer coming back	**398 (71.3%)**	100 (71.4%)	298 (71.3%)	0.975	21 (91.3%)	58 (74.4%)	21 (53.8%)	**0.005**	16 (69.6)	158 (76.3%)	124 (66.0%)	0.074
Coping with side-effects of treatment	**142 (25.4%)**	39 (27.9%)	103 (24.6%)	0.45	6 (26.1%)	24 (30.8%)	9 (23.1%)	0.667	11 (47.8%)	61 (29.5%)	31 (16.5%)	**<0.001**
Coping with disability due to surgery	**158 (28.3%)**	69 (49.3%)	89 (21.3%)	**<0.001**	13 (56.5%)	41 (52.6%)	15 (38.5%)	0.266	9 (39.1%)	50 (24.2%)	30 (16.0%)	**0.014**
Feeling isolated	**50 (9.0%)**	11 (7.9%)	39 (9.3%)	0.597	5 (21.7%)	5 (6.4%)	1 (2.6%)	**0.02**	7 (30.4%)	28 (13.5%)	4 (2.1%)	**<0.001**
Worrying about family and friends	**166 (29.7%)**	42 (30.0%)	124 (29.7%)	0.94	7 (30.4%)	31 (39.7%)	4 (10.3%)	**0.005**	14 (60.9%)	75 (36.2%)	35 (18.6%)	**<0.001**
Loss of control of my life	**111 (19.9%)**	33 (23.6%)	78 (18.7%)	0.208	8 (34.8%)	22 (28.2%)	3 (7.7%)	**0.018**	10 (43.5%)	53 (25.6%)	15 (8.0%)	**<0.001**
The possibility of dying	**156 (28.0%)**	40 (28.6%)	116 (27.8%)	0.852	9 (39.1%)	26 (33.3%)	5 (12.8%)	**0.032**	12 (52.2%)	73 (35.3%)	31 (16.5%)	**<0.001**
Why I got this cancer	**149 (26.7%)**	35 (25.0%)	114 (27.3%)	0.599	5 (21.7%)	23 (29.5%)	7 (17.9%)	0.367	5 (21.7%)	61 (29.5%)	48 (25.5%)	0.564
Spiritual issues	**11 (2.0%)**	3 (2.1%)	8 (1.9%)	0.866	0 (0%)	2 (2.6%)	1 (2.6%)	0.74	2 (8.7%)	5 (2.4%)	1 (0.5%)	**0.02**
Concerns about money	**73 (13.1%)**	25 (17.9%)	48 (11.5%)	0.053	4 (17.4%)	20 (25.6%)	1 (2.6%)	**0.009**	6 (26.1%)	38 (18.4%)	4 (2.1%)	**<0.001**
Not getting support services	**33 (5.9%)**	11 (7.9%)	22 (5.3%)	0.26	1 (4.3%)	9 (11.5%)	1 (2.6%)	0.186	3 (13.0%)	18 (8.7%)	1 (0.5%)	**<0.001**
Something else	**45 (8.1%)**	10 (7.1%)	35 (8.4%)	0.644	2 (8.7%)	5 (6.4%)	3 (7.7%)	0.921	2 (8.7%)	16 (7.7%)	17 (9.0%)	0.894
**Clarity of follow-up information**												
Clear	**428 (84.9%)**	115 (86.5%)	313 (84.4%)		20 (90.9%)	60 (80.0%)	35 (97.2%)		17 (81.0%)	154 (82.4%)	142 (87.1%)	
Not clear	**63 (12.5%)**	15 (11.3%)	48 (12.9%)		1 (4.5%)	13 (17.3%)	1 (2.8%)		4 (19.0%)	27 (14.4%)	17 (10.4%)	
Not given information	**8 (1.6%)**	2 (1.5%)	6 (1.6%)		1 (4.5%)	1 (1.3%)	0 (0%)		0 (0%)	3 (1.6%)	3 (1.8%)	
Don't know	**5 (1.0%)**	1 (0.8%)	4 (1.1%)		0 (0.0%)	1 (1.3%)	0 (0%)		0 (0%)	3 (1.6%)	1 (0.6%)	
				0.944				0.162				0.756
**Aware of how to contact sarcoma team**	**482 (89.6%)**	123 (88.5%)	359 (90.0%)	0.621	20 (87%)	68 (88.3%)	35 (89.7%)	0.944	19 (86.4%)	179 (89.9%)	161 (90.4%)	0.834
**Sarcoma UK**												
Told about Sarcoma UK	**133 (24.3%)**	34 (24.3%)	99 (24.3%)	0.912	7 (30.4%)	21 (26.9%)	6 (15.4%)	0.327	6 (26.1%)	57 (28.2%)	36 (19.8%)	0.262
Given Sarcoma UK toolkit	**104 (19.0%)**	14 (10.1%)	90 (22.1%)	**0.003**	3 (13.0%)	6 (7.8%)	5 (12.8%)	0.827	3 (13.0%)	49 (24.1%)	38 (21.0%)	**0.035**
**Told about local support groups/charities**												
Generic cancer	**190 (35.8%)**	44 (32.4%)	146 (37.1%)	0.514	7 (30.4%)	23 (30.3%)	14 (37.8%)	0.832	6 (26.1%)	78 (39.2%)	62 (36.0%)	0.244
Sarcoma-specific	**92 (17.5%)**	21 (15.3%)	71 (18.2%)	0.561	4 (17.4%)	12 (15.8%)	5 (13.2%)	0.597	3 (13.0%)	40 (20.2%)	28 (16.6%)	0.321
**Websites used**												
Macmillan	**231 (41.4%)**	61 (43.6%)	170 (40.7%)	0.546	14 (60.9%)	41 (52.6%)	6 (15.4%)	**<0.001**	17 (73.9%)	97 (46.9%)	56 (29.8%)	**<0.001**
Cancer Research UK	**108 (22.4%)**	33 (23.6%)	75 (17.9%)	0.145	9 (39.1%)	22 (28.2%)	2 (5.1%)	**0.003**	10 (43.5%)	46 (22.2%)	19 (10.1%)	**<0.001**
Sarcoma UK	**86 (15.4%)**	20 (14.3%)	66 (15.8%)	0.67	3 (13.0%)	16 (20.5%)	1 (2.6%)	**0.032**	6 (26.1%)	38 (18.4%)	22 (11.7%)	0.073
BCRT	**11 (2.0%)**	8 (5.7%)	3 (0.7%)	**<0.001**	3 (13.0%)	5 (6.4%)	0 (0%)	0.094	1 (4.3%)	0 (0%)	2 (1.1%)	**0.048**
GIST support UK	**1 (0.2%)**	0 (0.0%)	1 (0.2%)	0.562	0 (0%)	0 (0%)	0 (0%)	n/a	0 (0%)	1 (0.5%)	0 (0%)	0.6
Other	**45 (8.1%)**	9 (6.4%)	36 (8.6%)	0.411	1 (4.3%)	6 (7.7%)	2 (5.1%)	0.786	3 (13%)	20 (9.7%)	13 (6.9%)	0.46
